# Association between BDNF rs6265 polymorphisms and postoperative cognitive dysfunction in Chinese Han Population

**DOI:** 10.1002/brb3.1800

**Published:** 2020-08-17

**Authors:** Songhui Xie, Lu Yu, Mingming Zhou, Li Liu, Daoyun Lei, Chao Han

**Affiliations:** ^1^ Department of Anesthesiology The Affiliated Yixing Hospital of Jiangsu University Yixing China; ^2^ Yixing Clinical College Medical College of Yangzhou University Yixing China

**Keywords:** BDNF, cognition, genotype, polymorphism, single nucleotide

## Abstract

**Introduction:**

Brain‐derived neurotrophic factor (BDNF) plays a critical role in the pathogenesis of postoperative cognitive dysfunction (POCD). In present study, we aimed to assess the possible association between POCD and BDNF rs6265 polymorphisms.

**Methods:**

124 patients aged 60 years or older scheduled for elective surgery under general anesthesia and 25 age‐ and gender‐matched healthy volunteers were recruited. POCD was identified using a neuropsychological test battery administered preoperatively, 7 days, and 3 months after surgery. Genotyping of rs6265 was performed using polymerase chain reaction amplification and restriction fragment length polymorphism analysis.

**Results:**

99 patients and 25 healthy controls were finally enrolled in the analysis. 29(29.3%) and 18(18.2%) of 99 patients had POCD at 7 days and 3 months after surgery, respectively. The patients carrying a G allele at the rs6265 locus showed a lower risk for POCD than an A allele carriers on postoperative 7 days, but not 3 months after surgery (OR = 0.67; 95% CI: 0.47–0.96; *p* = .017; OR = 0.69; 95% CI: 0.42–1.13; *p* = .14, respectively). The risk of POCD at 7 days following surgery was significantly lower in additive model (OR = 0.41; 95% CI: 0.2–0.84; *p* = .015) and dominant model (OR = 0.35; 95% CI: 0.13–0.96; *p* = .042).

**Conclusion:**

We tentatively demonstrate that BDNF rs6265 polymorphisms might be associated with occurrence of POCD at 7 days after surgery and the A > G mutant at the rs6265 locus be likely a protective factor for early POCD in Chinese Han population.

## Graphical abstract

We used SNPs to detect the genetic polymorphism of BDNF rs6265 in surgical patients. We tentatively demonstrate that BDNF rs6265 polymorphisms be associated with occurrence of POCD at 7 days after surgery and the GG allele at the rs6265 locus be likely a protective factor for early POCD in Chinese Han population.

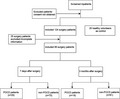

## 1 INTRODUCTION

Postoperative cognitive dysfunction (POCD), also named as postoperative neurocognitive disorder (NCD) (Evered, Silbert, & Knopman, 2018), is a highly prevalent disorder, especially in elderly patients, characterized by acute or persistent deficits in attention, concentration, learning, and memory following surgery, which can be detected by a battery of neuropsychological tests (Skvarc, Berk, & Byrne, 2018). POCD can prolong hospital stay, reduce quality of life, increase mortality, and aggravate the burden of public health, leading to significant clinical, social, and financial impacts on patients and their communities. The early ISPOCD1 study reported an incidence of POCD 25.8% one week and 9.9% three months after surgery in patients aged over 60 years who underwent major noncardiac surgery (Moller, Cluitmans, & Rasmussen, 1998), and POCD has become an increasingly important issue with extensive surgeries on older patients.

Although several risk factors for POCD have been identified (Sathananthan, 2018), the pathophysiological mechanism underlying POCD remains unclear. Accumulating evidences indicated the key role of hippocampal neuro‐inflammation in the disease process (Hovens et al., 2014; Terrando, Eriksson, & Ryu, 2011). Brain‐derived neurotrophic factor (BDNF) and its intra‐neuronal pathway have been implicated as mediators between neuro‐inflammation and neuronal dysfunction (e.g., decreased neurogenesis, synaptic plasticity, and LTP) (Barrientos et al., 2004; Yirmiya & Goshen, 2011). Many studies have shown that activation of BDNF signaling pathway could attenuate cognitive impairment after surgery and reduce the elevated levels of inflammatory cytokines (Chen, Wu, & Gu, 2018; Wei, Zheng, & Liu, 2018), suggesting the critical role of BDNF in the development of POCD.

Brain‐derived neurotrophic factor is the most ubiquitous and intensively studied member of the neurotrophic factor family, and the human BDNF gene located on chromosome 11 P13‐14 expresses at least three functional BDNF peptides (proBDNF, mBDNF, and the prodomain) that elicit independent biological effects (Notaras & Buuse, 2019). Although many single‐nucleotide polymorphisms in the BDNF gene are described, few studies on this topic were available except of the BDNF rs6265 polymorphism. Rs6265 is a missense mutation located in the exon of the BDNF gene and is present in approximately 30%–50% of the population (Shimizu, Hashimoto, & Iyo, 2004). The mutation of G to A results in an amino acid residue shift from valine (Val) to methionine (Met) at codon 66 within the BDNF prodomain, which interferes with a sortilin‐binding site disrupting in the intracellular trafficking and thus affecting the sorting of BDNF into secretory vesicles, resulting in reduction of activity‐dependent secretion (Notaras & Buuse, 2015). The rs6265 polymorphism has been found to be associated with memory performance (Kambeitz et al., 2012), Alzheimer disease (Huang, Huang, Cathcart, Smith, & Poduslo, 2007), Parkinson's disease (D'Souza & Rajkumar, 2019), traumatic brain injury (Giarratana, Teng, & Reddi, 2019), greater severity of CAD and incidence of CVD‐related clinical events (Jiang, Babyak, & Brummett, 2017), acute ischemic stroke (Zhou, Ma, & Fang, 2019), bipolar disorder (Lee, Wang, & Chen, 2016), major depression (Schumacher, Jamra, & Becker, 2005), and schizophrenia (Notaras & Buuse, 2015).

In present study, we investigated the correlations of BDNF polymorphism at the rs6265 locus with POCD in Chinese Han population.

## 2 PARTICIPANTS AND METHODS

### 2.1 Participants

After approval from the hospital ethics committee, a total of 124 patients scheduled for elective surgery under general anesthesia in the affiliated Yixing Hospital of Jiangsu University from September 2015 to December 2016 were enrolled, while 25 age‐ and gender‐matched healthy volunteers were recruited as controls for POCD calculation. The informed consents for genetic testing were obtained from all patients involved in this study. These patients met the following inclusion criteria: American Society of Anesthesiologists physical classification classes I–II, age 60 years or older, no history of symptomatic cerebrovascular disease, renal failure, active liver disease, bleeding disorders, or visual and hearing impairment. Exclusion criteria were as follows: refusal, pre‐existing neurological or clinically evident neurovascular disease, preoperative mini‐mental state examination (MMSE) scores less than 24, duration of surgery less than 2 hr, and unable to complete all of neuropsychological (NP) tests, and those who have serious postoperative complications and obvious organic change of brain on CT examination were eliminated as well.

### 2.2 Collection of clinical information

Demographic data, such as age, sex, weight, height, education level, along with history of hypertension, diabetes, smoking, and drinking, were collected by an arranged anesthesiologist. Peri‐operative risk factors, including the type of surgery and anesthesia, analgesics, anticholinergic drug, duration of surgery, events of hypotension during operation, and blood loss, were also recorded.

### 2.3 Neuropsychological assessment

All patients and controls completed a battery of six NP tests conducted by trained interviewers. The test battery consisted of Visual Verbal Learning, Concept Shifting Task, Stroop Color Word Test, Memory Scanning Task, Letter‐Digit Coding, and Reaction time testing with the Four Boxes Test (Rasmussen, Houx, Skovgaard, Hanning, & Moller, 2001). The patients received the test battery within the day prior to surgery, then on day 7, and at three months after surgery, while the control volunteers underwent NP testing at timepoints corresponding to assessments in the patients undergoing surgery. Each NP test was scored individually for surgery and control subjects, then calculated a change score by subtracting the preoperative score from the 7 day and 3 month score. When a change score was at least 1.96 *SD* lower than the mean score of the matched control group after adjusting for expected change over time using controls, we considered the test “positive.” POCD was defined using the reliable change index as when two or more of the six baseline tests were “positive.”

### 2.4 Collection of blood and extraction of DNA

Fasting peripheral venous blood samples (5 ml) were collected from superficial veins of the upper extremity at the elbow of each patient in the morning, with EDTA acid as the anticoagulant. The samples were then stored at −20°C until genotyping.

### 2.5 Genotyping

Genomic DNA was extracted from the blood samples using DNA extraction kit (TIANGEN Co., China). Genotyping of rs6265 was determined by polymerase chain reaction amplification and restriction fragment length polymorphism analysis, as described elsewhere (Zhou et al., 2019).

### 2.6 *Statistical* analysis

Statistical analyses were conducted using SPSS 19.0 (SPSS Inc., Chicago, USA). Continuous variables and categorical variables are described as means ± standard deviation and numbers or percentages appropriately. Categorical variables were analyzed by the chi‐square (*χ* ^2^)‐test, and the continuous variables was analyzed by independent *t* test. The association between the genotypes and risk of POCD was assessed by calculating values of odds ratios (ORs) and 95% confidence intervals (95% CIs). Hardy–Weinberg equilibrium (HWE) value was calculated by chi‐squared test.

## 3 RESULTS

**Figure 1:**
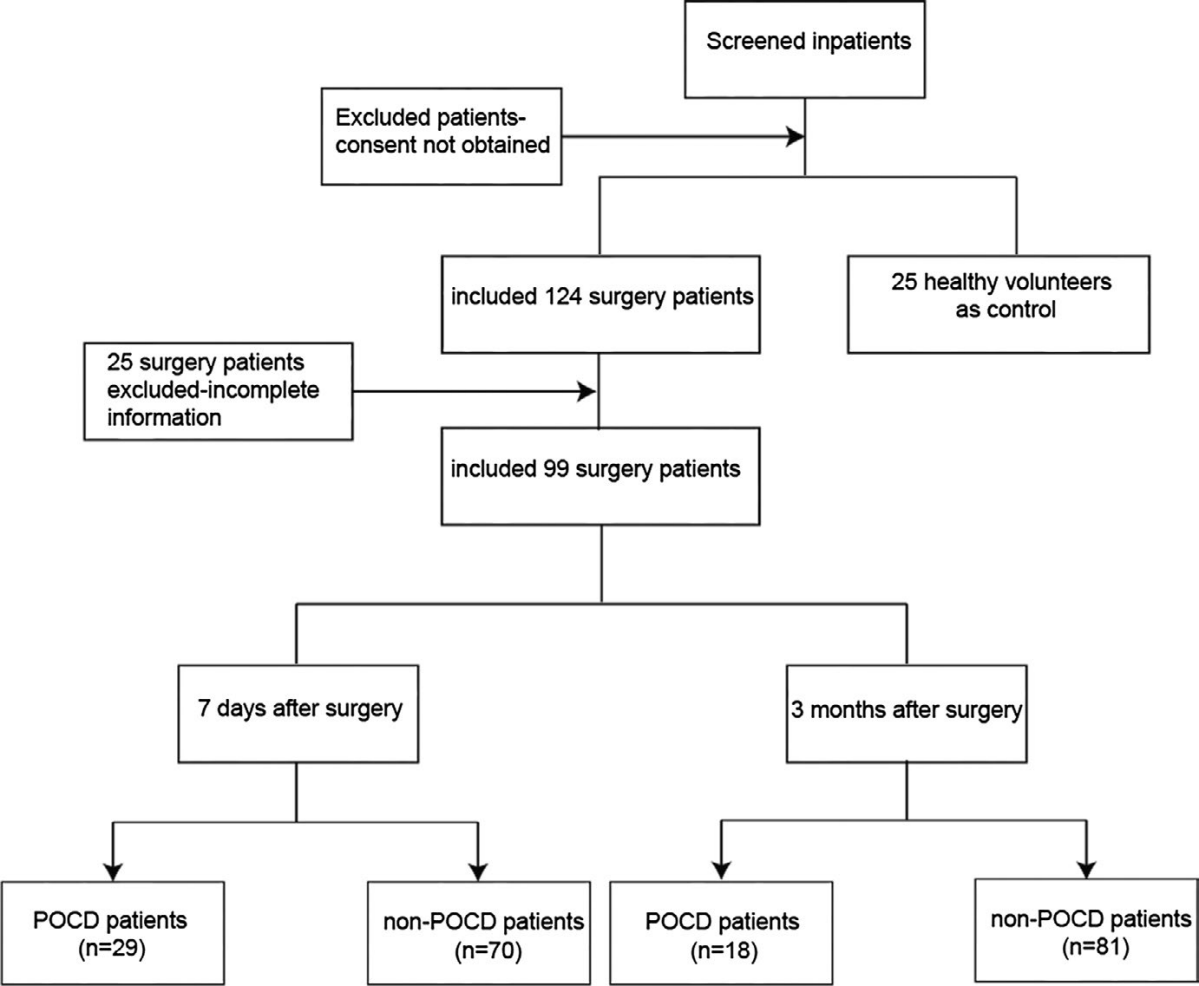
study flow chart

As described above, 124 patients were recruited in this study. Of the 124 patients, 25 were excluded after surgery for the following reasons: duration of surgery less than 2 hr, death within 3 months, inability to complete NP test because of postoperative complications or intensive care unit admission, and loss of follow‐up at 3 months. A total of 99 patients were finally enrolled in the analysis. According to the POCD definition, of 99 patients, 29(29.3%) and 18(18.2%) have been found to have POCD at 7 days and 3 months after surgery, respectively. The study flow chart is shown in Figure 1.

The demographic data between surgical patients and healthy volunteers were compatible (age, 71.7 ± 4.5 versus 68.2 ± 3.1; sex 79.8% versus 76.0%; BMI, 25.5 ± 2.3 versus 24.6 ± 1.4; hypertension, 47.5% versus 44.0%; diabetes, 12.1% versus 12.0%; smoking, 42.4% versus 36.0%; drinking, 37.4% versus 32.0%; education, 4.8 ± 2.2 versus 5.6 ± 2.3). The demographic and clinical data of POCD and NO‐POCD patients 7 days and 3 months after surgery were shown in Table 1. No significant difference was available between the two groups except of education and recovery time at postoperative 3 months.

**Table 1:** Demographic and clinical data of POCD and NO‐POCD patients 7 days and 3 months after surgery

Characteristic	Postoperative 7 days		*p*	Postoperative 3 months		*p*
	POCD (*n* = 29)	NO‐POCD (*n* = 70)		POCD (*n* = 18)	NO‐POCD (*n* = 81)	
Age (y)	72.6 ± 4.6	71.3 ± 4.1	.28	73.5 ± 3.7	72.2 ± 4.4	.24
Sex (male)	23 (79.3)	56 (80.0)	.93	15 (83.3)	64 (79.0)	.67
BMI	25.6 ± 2.1	25.2 ± 2.3	.42	25.4 ± 1.9	25.5 ± 2.2	.85
Hypertension	14 (48.3)	33 (47.1)	.91	9 (50.0)	38 (46.9)	.81
Diabetes	3 (10.3)	9 (12.9)	.72	2 (11.1)	10 (12.3)	.88
Smoking	11 (37.9)	31 (44.3)	.56	9 (50.0)	33 (40.7)	.47
Drinking	10 (34.5)	27 (38.6)	.70	8 (44.4)	29 (35.8)	.49
Education (y)	4.3 ± 2.1	5.1 ± 1.8	.07	4.1 ± 1.7	5.2 + 1.7	.02
Type of surgery						
Abdominal	18 (62.1)	32 (45.7)		12 (66.7)	38 (46.9)	
Thoracic	7 (24.1)	18 (25.7)		4 (22.2)	21 (25.9)	
Orthopedics	3 (10.3)	15 (21.4)		2 (11.1)	16 (19.8)	
Others	1 (3.4)	5 (7.1)	.39	0 (0)	6 (7.4)	.36
Type of anesthesia						
Propofol Intravenous	13 (44.8)	43 (61.4)		8 (44.4)	48 (59.3)	
Sevoflurane Inhalation	16 (55.2)	27 (38.6)	.12	10 (55.6)	33 (40.7)	.25
Anticholinergic	24 (82.8)	57 (81.45)	.87	16 (88.9)	65 (80.2)	.38
Duration of surgery (hr)	2.7 ± 0.4	2.5 ± 0.6	.11	2.8. ±0.4	2.6 ± 0.5	.11
Events of hypotension during operation	11 (37.9)	23 (32.9)	.62	8 (44.4)	26 (32.1)	.31
Blood loss (ml)	353.8 ± 45.2	337.5 ± 55.4	.16	362.5 ± 62.7	346.4 ± 51.3	.25
Recovery time (min)	26.6 ± 6.3	25.2 ± 6.6	.33	28.5 ± 7.4	24.8 ± 6.2	. 03
Pain scores within 24 hr after surgery	3.3 ± 1.4	2.8 ± 1.3	.09	3.4 ± 1.6	2.7 ± 1.4	.07

Note: (Percentage).

Rs6265 genotypes/allele frequencies distribution and association with POCD are shown in Table 2. HWE for rs6265 genotypes reached the equilibrium level, HWE (chi‐squared test = 4.402, *p* = .11).

**Table 2:** Rs6265 genotypes and allele frequencies between POCD and NO‐POCD

	Group	Allele		OR (95% CI)	*p*	genotype			Additive model		Dominant model		Recessive model	
		A	G			AA	AG	GG	OR (95% CI)	*p*	OR (95% CI)	*P*	OR (95% CI)	*p*
7 days	POCD	36	22			10	16	3						
	NO‐POCD	61	79	0.67 (0.47–0.96)	.017	11	39	20	0.41 (0.2–0.84)	.015	0.35 (0.13–0.96)	.042	0.29 (0.078–1.06)	.061
3 months	POCD	28	22			8	12	5						
	NO‐POCD	69	79	0.69 (0.42–1.13)	.14	13	43	18	0.65 (0.32−0.302)	.22	0.45 (0.16–1.27)	.13	0.77 (0.25–2.37)	.65

## 4 DISCUSSION

As gene technology is widely used to detect the susceptibility of a certain disease, anesthesiologists have been committed to find genetic indicators of POCD susceptibility to respond to the adoption of positive anesthesia nursing. Tardiff et al (Tardiff et al., 1997). first reported the APOE‐ε4 genotype related to cognitive dysfunction after cardiopulmonary bypass. Subsequent studies presented conflicting results (Abildstrom, Siersma, & Rasmussen, 2004; Bryson et al., 2011; Cai et al., 2012; Heyer et al., 2005; McDonagh et al., 2010). A recent cohort analysis of 1,033 participants found that older men with APOE‐ε4 allele may be more vulnerable to postoperative cognitive dysfunction than older women with APOE4 allele (Schenning, Murchison, Mattek, Kaye, & Quinn, 2019). Up to date, the relation between APOE‐ε4 and POCD is still unclear. Some other studies investigated candidate genes regulating biologic pathways for inflammation, cell matrix adhesion/interaction, coagulation‐thrombosis, lipid metabolism, vascular reactivity, and drug metabolism which are associated with incidence of POCD (Mathew, Podgoreanu, & Grocott, 2007; Steinmetz et al., 2012), suggesting P‐selectin and CRP genes in modulating susceptibility to cognitive decline after cardiac surgery.

In this study, for the first time, we studied the correlations between polymorphism at the BDNF rs6265 (A/G) locus and POCD after surgery 7 days and 3 months. We discovered that patients carrying a G allele at the rs6265 locus showed a lower risk for POCD than an A allele carriers on postoperative 7 days, but not 3 months after surgery (OR = 0.67; 95% CI: 0.47–0.96; *p* = .017; OR = 0.69; 95% CI: 0.42–1.13; *p* = .14; 7 days and 3 months after surgery, respectively). Additionally, the additive model showed that risk of POCD in carriers of the GG and AG genotypes was significantly lower than AA genotype individuals in 7 days following surgery (OR = 0.41; 95% CI: 0.2–0.84; *p* = .015). The dominant model also showed the same trend (OR = 0.35; 95% CI: 0.13–0.96; *p* = .042). These results suggested that BDNF rs6265 A > G mutant might be an independent protective factor for POCD at 7 days after surgery.

Previous study has shown that inhibition of the brain‐derived neurotrophic factor signaling pathway in the hippocampus and amygdala contribute to surgical incision‐induced postoperative cognitive function impairment in mice (Liu, Liu, Ma, & Zhao, 2018), indicating the pivotal role of BDNF in POCD. BDNF gene may affect BDNF secretion and/or function, thereby affecting postoperative cognitive function. In a human study, BDNF val66met carriers exhibited relatively diminished hippocampal engagement in comparison with val homozygotes during both encoding and retrieval processes, and interaction between the BDNF val66met genotype and the hippocampal response during encoding accounted for 25% of the total variation in recognition memory performance, suggesting that the BDNF66Met substitution may alter the encoding of engrams (Hariri et al., 2003). Furthermore, in a BDNF Val66Met knock‐in mouse model BDNF Met/Met mice were found to have deficient contextual fear memory as well as reduced hippocampal volumes relative to BDNF Val/Val mice, and the neurons isolated from this mouse model also exhibited defective activity‐dependent release of BDNF (Chen, Jing, & Bath, 2006). In addition, several other reports have also found that the BDNF 66Met allele also disrupts hippocampus‐dependent spatial memory on the water‐ and T‐mazes (Wang, Tian, & Dong, 2014; Yu et al., 2012). These evidences above might explain the underlying pathogenesis of POCD due to rs6265 polymorphism.

There are some limitations in our study that should be noted. First, this study is a single‐center study with a small sample size limited by fund and time. Second, this study only included the Han population in eastern China, and the findings need to be further verified in different ethnic populations in different regions. Third, our study included all kinds of surgeries and ignored the potential difference of POCD incidence between different operations. Lastly, we did not detect the changes of BDNF in plasma or cerebrospinal fluid BDNF which might partly reflect the differences of BDNF levels due to rs6265 polymorphism in the human brain.

Taken together, we tentatively suggest that rs6265 genotypes might be associated with the development of early POCD and the A > G mutant GG allele at the rs6265 locus is likely a protective factor for early POCD in Chinese Han population. However, it is clearly important that an independent study in large, well‐characterized samples need to replicate this potentially important finding.

## DISCLOSURE

The authors report no conflicts of interest in this work.

## AUTHOR CONTRIBUTION

Songhui Xie and Chao Han performed the research, Lu Yu selected participants contributed clinical information, Mingming Zhou and Li Liu analyzed the data, Chao Han designed the research study, and Daoyun Lei wrote the paper.

### Peer Review

The peer review history for this article is available at https://publons.com/publon/10.1002/brb3.1800.

## Data Availability

The data that support the findings of this study are available on request from the corresponding author. The data are not publicly available due to privacy or ethical restrictions.
